# Temporal Trends in Vertebral Dimensions – a case study from Finland

**DOI:** 10.1038/s41598-020-58340-9

**Published:** 2020-01-31

**Authors:** Niina Korpinen, Petteri Oura, Tiina Väre, Markku Niskanen, Jaakko Niinimäki, Jaro Karppinen, Juho-Antti Junno

**Affiliations:** 1 Faculty of Humanities, Department of Archaeology, University of Oulu, Oulu, Finland; 2 Medical Research Center Oulu, Oulu University Hospital and University of Oulu, Oulu, Finland; 3 Faculty of Medicine, Center for Life Course Health Research, University of Oulu, Oulu, Finland; 4 Faculty of Medicine, Research Unit of Medical Imaging, Physics and Technology, University of Oulu, Oulu, Finland; 5 Finnish Institute of Occupational Health, Oulu, Finland; 6 Faculty of Medicine, Cancer and Translational Medicine Research Unit, University of Oulu, Oulu, Finland

**Keywords:** Archaeology, Evolutionary developmental biology

## Abstract

Vertebral fractures and other back problems represent a major, increasing worldwide health problem. This has increased the need to better understand the reasons behind this phenomenon. In addition to a reduction in bone mineral density and overall size of the vertebral body, research has indicated a possible association between the shape of the endplate and spinal disorders. As one previous study has shown changes in vertebral body dimensions between contemporary people and their medieval counterparts, we wanted to examine the potential temporal trends in vertebral size and dimensions in Finnish samples of archaeological and contemporary individuals. To conduct this study, we utilized three archaeological populations from the 16^th^–19^th^ century and clinical materials from two population-based Finnish birth cohorts. As the average height of people has increased greatly since the first time period, we also height-adjusted the dimensions to provide a clearer picture of the dimensional changes that have occurred in the later temporal group. Our results were in agreement with those of the earlier study. The archaeological samples had a larger vertebral size than the contemporary population when height was adjusted for. Vertebral mediolateral width in particular had decreased, and the shape of the vertebral body had changed.

## Introduction

Back problems, particularly low back pain, have become major health issues across the world^1–3^. The 2010 Global Burden of Diseases (GBD) study found low back pain to be the greatest contributor to global disability in over half of the regions it studied. For all regions, it ranked in the top four leading causes of years lived with disability (calculated as YLDs)^2,4^. Hoy *et al*.^3^ estimated the global prevalence of activity-limiting low back pain that lasted longer than one day to be 11.9 ± 2.0% and the one-month prevalence to be 23.2 ± 2.9%. Back problems are frequent also in Finland, where over 40% of men and women report having experienced back pain in the last 30 days^5^. The 2017 GBD study as well ranked low back pain as the top contributor to years lived with disability in Finland, representing 10.6% of total YLDs^6^.

As back pain often accompanies different spinal ailments such as osteoporotic fractures and disc herniation, which themselves are growing problems in modern sedentary societies, finding the risk factors that contribute to them has become an important area of study. A number of studies have found that in addition to bone density, the size and shape of the vertebral body plays an important role in the vertebrae’s strength and biomechanics. For example, smaller vertebral bodies or cross-sectional areas have been found to correlate with osteoporotic fractures^7–10^. Smaller bones appear to experience greater stress during axial compression and further greater stress when exposed to bending forces^7,11^. Single dimensions of vertebral bodies have also shown to impact facture risk. Both greater anterior-posterior depth and smaller mediolateral width has been connected to an increased risk of vertebral fractures^8,12^. Furthermore, anterior-posterior depth has been reported to play a significant role in bending rigidity and in the relationship between axial and bending rigidity^13^.

The size and shape of the vertebral body endplate has also been associated with both disc herniations and Schmorl’s nodes^14–17^. Using the vertebral endplate is an indicator of intervertebral disc and viewing the discs as liquid-filled tubes, it was proposed that according to Laplace’s law, the discs’ ability to resist tensions decreases as the radius increases^14–16^. Therefore, a rounder shaped intervertebral disc could experience herniation more often in its posterior part, which could cause compression of the neural roots^14^.

Since these previous studies show indications that the size and shape of the vertebral body could be important factors in spinal health and biomechanics, we need to understand how internal and external factors influence human vertebral variation. A previous study by Junno *et al*.^18^ found that contemporary peoples’ vertebrae were significantly mediolaterally smaller but craniocaudally longer than those of their medieval counterparts. However, the study’s sample consisted of individuals from different populations and geographical locations, as the medieval samples were from Great Britain and Sweden and the contemporary sample was from Finland. As there are indications that differences in vertebral morphology might exist within the European population^19^, we wanted to clarify whether these differences found in the study truly were caused by temporal changes or if they in fact represent differences between populations. To investigate if similar trends could be detected in Finnish archaeological material, we utilized a sample of 42 specimens from three locations in Finland, which date mainly from the 16^th^–19^th^ century^20–24^, and two large contemporary cohort samples from Northern Finland^25,26^.

We decided to focus on the same second lowest lumbar vertebra as Junno *et al*.^18^ and several previous studies^27–33^. We chose this vertebra partly for easier comparison to previous results but also because the lower thoracic and lumbar sections of the spine are usually more vulnerable to osteoporotic fractures and other pathologies^34,35^. Therefore changes in this specific area could have a large impact on spinal health. Like the previous study^18^, we concentrated on changes in both the single dimensions and the overall size of the vertebral body. However, due to the possible role of the shape of the vertebral body in spinal ailments, we also wanted to examine potential temporal changes in the shape of the vertebral bodies. We used the ratio between mediolateral width and anterior posterior depth as an indicator of the shape of the vertebral body.

On the basis of previous results^18^, we hypothesized that there would be a decrease in the mediolateral width of the vertebral bodies due to the continuing changes in lifestyle and increase in craniocaudal lengths of the vertebral bodies, as a result from the increase in average height in the Finnish population over the last 100 years^36^.

## Results

The comparison of the raw unadjusted dimensions between archaeological sample and the contemporary 46-year-olds sample seemed to indicate a temporal increase in most dimensions (p < 0.005). However, no difference was found in cross-sectional area (CSA) in males (p = 0.713) or mediolateral (ML) width in females (p = 0.627). ML width had instead decreased (p = 0.004) in the contemporary males compared to their archaeological counterparts. (Table 1.). The dimensions of the contemporary 20-year-olds were smaller (p < 0.001) or similar (p > 0.05) with the archaeological ones. Only the CC height that had increased in the contemporary males (p < 0.001).

**Table 1:** Stature and vertebral dimensions in archaeological and contemporary samples, with crude and height-adjusted comparisons between samples.

Variable and sample	Males					Females				
	Mean value ± standard deviation	Crude comparison		Stature-adjusted comparison		Mean value ± standard deviation	Crude comparison		Stature-adjusted comparison	
		P value	*n*	P value	*n*		P value	*n*	P value	*n*
**Stature (cm)**										
Archaeological	166.9 ± 5.8	Ref.	24	—	—	155.7 ± 5.0	Ref.	18	—	—
Modern 20 y	175.3 ± 7.2	<**0**.**001**	143	—	—	163.9 ± 6.0	<**0**.**001**	217	—	—
Modern 30 y	—	—	—	—	—	—	—	—	—	—
Modern 46 y	178.7 ± 6.2	<**0**.**001**	616	—	—	164.8 ± 5.8	<**0**.**001**	742	—	—
**Vertebral CSA (mm**^2^**)**										
Archaeological	1496.4 ± 241.3	Ref.	22	Ref.	22	1108.3 ± 198.1	Ref.	18	Ref.	18
Modern 20 y	1330.7 ± 185.5	<**0**.**001**	143	<**0**.**001**	143	1061.3 ± 125.9	0.199	217	<**0**.**001**	217
Modern 30 y	1443.9 ± 188.6	0.268	144	—	—	1152.0 ± 130.9	0.232	227	—	—
Modern 46 y	1512.9 ± 214.0	0.713	616	<**0**.001*	616	1210.2 ± 158.8	**0**.**004**	742	0.742	742
**Vertebral CC height (mm)**										
Archaeological	26.5 ± 1.8	Ref.	24	Ref.	24	26.1 ± 1.4	Ref.	18	Ref.	18
Modern 20 y	29.0 ± 1.8	<**0**.**001**	143	<**0**.**001**	143	26.7 ± 1.5	0.125	217	0.289	217
Modern 30 y	29.7 ± 1.8	<**0**.**001**	144	—	—	27.3 ± 1.5	**0**.**001**	227	—	—
Modern 46 y	29.4 ± 1.6	<**0**.**001**	616	<**0**.**001**	616	27.7 ± 1.6	<**0**.**001**	742	0.098	742
**Vertebral ML width (mm)**										
Archaeological	53.8 ± 4.5	Ref.	23	Ref.	23	46.4 ± 3.8	Ref.	18	Ref.	18
Modern 20 y	49.4 ± 4.1	<**0**.**001**	143	<**0**.**001**	143	44.1 ± 3.0	**0**.**004**	217	<**0**.**001**	217
Modern 30 y	51.1 ± 3.9	**0**.**004**	144	—	—	45.9 ± 2.9	0.489	227	—	—
Modern 46 y	51.3 ± 4.1	**0**.**004**	616	<**0**.**001**	616	46.1 ± 3.4	0.627	742	**0**.**001**	742
**Vertebral AP depth (mm)**										
Archaeological	35.2 ± 3.3	Ref.	23	Ref.	23	30.2 ± 3.4	Ref.	18	Ref.	18
Modern 20 y	34.2 ± 2.4	0.107	143	<**0**.**001**	143	30.5 ± 2.1	0.518	217	**0**.039*	217
Modern 30 y	35.8 ± 2.4	0.271	144	—	—	31.9 ± 2.1	**0**.**003**	227	—	—
Modern 46 y	37.4 ± 2.8	<**0**.**001**	616	0.990	616	33.3 ± 2.4	<**0**.**001**	742	**0**.**003**	742
**Vertebral ML width/AP depth ratio**										
Archaeological	1.52 ± 0.11	Ref.	22	Ref.	22	1.55 ± 0.11	Ref.	18	Ref.	18
Modern 20 y	1.45 ± 0.11	**0**.**001**	143	**0**.**001**	143	1.45 ± 0.09	<**0**.**001**	217	<**0**.**001**	217
Modern 30 y	1.43 ± 0.10	<**0**.**001**	144	—	—	1.44 ± 0.09	<**0**.**001**	227	—	—
Modern 46 y	1.38 ± 0.09	<**0**.**001**	616	<**0**.**001**	616	1.38 ± 0.09	<**0**.**001**	742	<**0**.**001**	742

Ref. = Reference category.

*after height adjustment, dimension was smaller among the contemporary humans than among the archaeological individuals, even though the raw value was larger.

(CC) craniocaudal, (ML) mediolateral and (AP) anterior-posterior.

The vertebrae of contemporary males (age of 46) showed an average increase in CSA of 16.5 mm^2^, 2.9 mm in craniocaudal (CC) height, and 2.2 mm in anterior-posterior (AP) depth. The vertebrae of contemporary females (age of 46) exhibited an average increase of 101.9 mm^2^ in CSA, 1.6 mm in CC height and 3.1 mm in AP depth. In contrast, mediolateral (ML) width seemed to have decreased by 2.5 mm among the contemporary males and remained almost the same among the females with only an 0.3 mm decrease in the contemporary population.

As the average stature had increased from archaeological to modern times, with males showing an increase of 11.8 cm and females showing increase of 9.1 cm (Table 1), we used general linear model to adjust the dimension to the height. The height-adjusted results showed that, the contemporary males (at the age of 46) had smaller vertebral ML width (p < 0.001) and CSA (p < 0.001), but their CC height (p < 0.001) was greater than the archaeological individuals. Similar results were obtained for the 20-year-olds (p < 0.001 for all). In terms of AP depth, the 20-year-olds were smaller than their archaeological counterparts (p < 0.001) but the 46-year-olds were of the same size (p = 0.990).

The height-adjusted results showed that the contemporary females had smaller ML width (20-year-olds p < 0.001 and 46-year-olds, p = 0.001) than the archaeological specimens. They presented no significant difference in CC height (20-year-olds, p = 0.289 and 46-year-olds, p = 0.098) or in CSA (46-year-olds, p = 0.742). CSA was larger in the archaeological specimens only, in comparison with the younger contemporary sample (p < 0.001). The both contemporary samples showed larger AP depth (20-year-olds, p = 0.039 and 46-year-olds, p = 0.003) than the archaeological sample.

**Figure 1:**
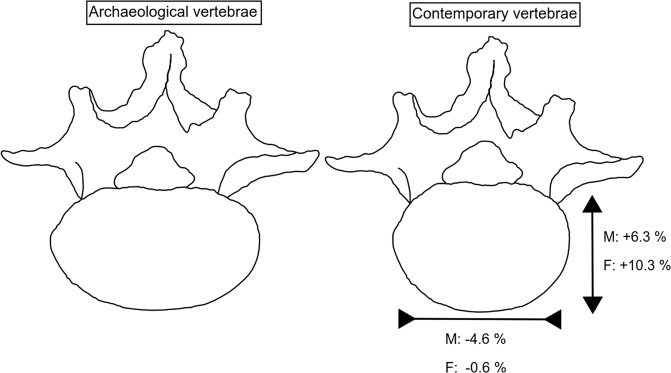
Picture illustrating the difference in vertebral body shape between contemporary and archaeological individuals. The arrow indicates the increase in AP depth and reversed arrow indicates the decrease in ML width. Next to arrows are the average percentual changes in the dimensions in the 46-years-old contemporary males (M) and females (F) sample compared to the archaeological sample.

To study how the shape of the vertebral body varied between the samples we compared the ratio of the ML width and AP depth between contemporary and archaeological samples. The unadjusted ratio was greater in the archaeological sample compared to contemporary people (1.52 ± 0.11 versus 1.38 ± 0.09 among the males and 1.55 ± 0.11 versus 1.38 ± 0.09 among the females), and even after adjustment for height, the difference remained clear (both sexes, p < 0.001). This indicates that the ratio between ML width and AP depth in the contemporary population has decreased. As such it would seem that contemporary people have slightly rounder-shaped vertebral bodies then the archaeological individuals, which suggests temporal change in vertebral body shape (see Fig. 1.).

## Discussion

The results were mostly in line with those of the earlier study^18^. ML width in particular decreased significantly in the contemporary males and was relatively smaller in the females. We also detected CC height increase within both sexes. Interestingly, only males showed a statistically significant increase in CC height after the values were height-adjusted. CSA had decreased among the males but remained similar among the females when adjusted for height. However, in contrast to the earlier study^18^, our results indicated an increase in AP depth, especially within the contemporary females, who still showed a significant difference after height adjustment. Taking these into consideration, it is not surprising that the ratio between vertebral ML width and AP depth had greatly decreased in the contemporary groups in comparison to the archaeological groups. Interestingly the change in the ratio was quite similar among both sexes, although it looks like the reasons may be different, as males experienced a decrease in ML width and females an increase in AP depth. The ML width/AP depth ratio also seems to indicate that the overall shape of the vertebrae has become rounder, with less difference between ML width and AP depth.

This could be a worrying trend, as there are indications that a decrease in the ratio between the ML width and AP depth of the vertebral body could increase the change of degeneration of the intervertebral discs. Harrington and collegues^14^ concluded that shape had a stronger association with disc herniation than the size of the vertebral endplate, especially among larger males. They utilized Laplace’s law to demonstrate that a slightly more ovoid endplate could more often lead to failure in lateral sectors of the disc and result in protection from nerve root compression. In a sense, this decrease in AP radius could be achieved by either decreasing AP depth or increasing ML width. Using geometric morphometrics, Plomp *et al*.^15,17^ also found that a rounder vertebral endplate was associated with Schmorl’s nodes; depressions in either or in both inferior and superior surface of the vertebral body caused by vertical herniation of the nucleus pulposus through annulus fibrosus. Especially a rounder posterior part of the endplate was typical feature for the individuals with Schmorl’s nodes. This would support the idea that a smaller difference between width and depth dimensions could lead to intervertebral disc herniation.

The less round-shaped vertebral body shape might also offer protection from vertebral fractures, as Ross *et al*.^12^ found that females with greater AP depth were at a higher risk of vertebral fractures. Similarly, Vega *et al*.^8^ found that males with vertebral fractures tended to have smaller ML width. Together these seem to indicate that the risk is likely linked to change in the vertebral body shape or ratio between the width and depth, rather than simply vertebrae being narrower or deeper, as changing either dimension would cause a change in overall shape of the vertebral body. Thus, if less round-shaped vertebral bodies are more protective of the intervertebral disc and could be biomechanically more advantageous, why does it seem that the trend we see in our results is completely opposite in the contemporary lumbar vertebrae?

Junno and colleagues^18^ speculated that the decrease in physical activity from medieval times to the modern day could partly explain the decreasing ML width. However, more recent research^27,28^ on contemporary people has indicated that higher activity at a younger age does not influence the size or strength of the vertebrae in older age. On the other hand, these studies^27,28^ have focused on sports and hobbies that are not practised all day, every day, all year round. In the 16^th^–19^th^ centuries, people started working at a very young age, even under the age of 10, and days were long and physically hard. Peasants in particular had to work from morning to evening all year round to survive^37–39^. This continuous, strenuous physical activity could have affected vertebral dimensions to a much greater extent during childhood and over later years than we can observe in contemporary physically active people. Thus, we cannot rule out the possibility of physical activity being a factor in these changes.

In addition to changes in physical activity, another great change experienced by the later temporal group is an approximately 10 cm height growth. This stature increase is mostly likely linked to improvement in diet^40,41^, as prior to the mid-19^th^ century, Finland experienced multiple famines and the diversity and quality of food was rather poor, especially in the lower economical classes^37,42^. This means that many people were suffering some sort of malnourishment and nutrient deficiency^37^ that could have affected their growth. As diet has improved in the last 100 years, the average height has increased in Finnish population^36^. Although the majority of the late human height increases is documented as having occurred in the length of the legs^43,44^, there are also indications that increase in sitting height does partly contribute the height increase^44^. We observed a clear increase in the craniocaudal height of the vertebrae in our contemporary humans, which we suspect to be related to height increase as the previous study has already demonstrated relationship between the vertebral height and stature^18^. It is, however, unclear how the height increase of the vertebral body affects the proportion of other vertebral dimensions. Should we expect the whole vertebra to grow on an isometric scale? Our results do not seem to support this, as ML width had decreased among males in particular. AP depth on the other hand had stayed relatively similar in males and slightly increased in females. One reason for this might be the need to try and maintain relatively similar CSA even as the ML width has decreased.

As very little research seems to have focused on the possible impacts that the shape of the vertebral body or the ratio between AP depth and ML width could have on the probability of fracturing one’s vertebrae, it is hard to determine which might be more important in fractures; plain size, shape, or both. In intervertebral disc herniation instead, it seems that shape could make a difference. Either way, our results would seem to indicate both a decrease in the relative size of the vertebral body and a change in shape which, together, could have a large impact on spinal health. Therefore, further studies on the effects of vertebral body shape on the biomechanical properties of the vertebra and on the intervertebral disc health are needed.

Our results indicated temporal changes in the size and shape of the vertebral body. Although the actual size of the vertebral body, apart from ML width, has increased among contemporary people, when stature increase is taken into consideration, the relative size of the vertebral body has actually decreased. In addition, to the change in size, the ratio between ML width and AP depth indicates that the shape of vertebral bodies has also changed, especially due to the decrease in ML width. Either change could have considerable consequences for spinal health, as previous studies^7–12,14–17^ have connected both features to spinal problems.

## Methods

Our study sample consisted of 42 archaeological specimens from three different locations in Finland. Fifteen individuals (11 males and 4 females) were from the cemetery of the Church of St Jacobs, located in Renko in inland southern Finland. This cemetery dates between the 16^th^ and 19^th^ century and is a rural site^21,23^. Twelve individuals (8 males and 4 females) were from the burial ground of the Porvoo Cathedral, located in the town of Porvoo on the southern coast of Finland. This site dates between the 14^th^ and 18^th^ centuries, but it was suspected that the majority of the individuals dated to the 17^th^ and 18^th^ century because they had been buried in coffins^20,23^. Porvoo is one of the oldest towns in Finland and was one of the largest in the mid-18^th^ century. Fifteen individuals (5 males and 10 females) were from the church yard of the Holy Trinity Church in Rauma and were estimated to date mainly between the 18^th^ and 19^th^ century^22,24^.

Our modern human samples consisted of two population-based Finnish birth cohorts (NFBC1966^25^ and NFBC1986^26^). The cohorts were comprised of individuals born in Northern Finland, i.e. in the provinces of Oulu and Lapland, in 1986 and 1966. Initially, the NFBC1966 and NFBC1986 comprised 12 058 and 9432 individuals, respectively. Over time, both cohort populations have been followed up closely at regular intervals. The cohorts are administered by the NFBC Project Center (http://www.oulu.fi/nfbc/).

In this study, we utilized representative MRI-scanned subsamples from both cohorts. The NFBC1986 population had lumbar MRI scans available from the ages of 20 and 30 years (n = 375 individuals with no vertebral pathologies)^45^, and NFBC1966 had scans from the age of 46 (n = 1363 individuals with no vertebral pathologies)^46^. Data on sex and stature were available via additional cohort data collections; sex data were available for all the modern samples, but stature was available for only the 20- and 46-year-old samples.

The study was conducted in accordance with the Declaration of Helsinki and approved by the Ethical Committee of the Northern Ostrobothnia Hospital District in Oulu, Finland. All the cohort members provided written informed consent to the study. We encrypted all personal identity information and replaced it with identification codes, providing full anonymity for the whole cohort study population.

From the archaeological sample, we took four osteological measurements of the superior endplate of each vertebra, using standard or digital callipers. These included: anterior height, posterior height, maximum mediolateral (ML) width, and maximum anterior-posterior (AP) depth. We took measurements from the vertebral body and recorded them to the closest 0.5 mm or 0.01 mm (digital). We also measured the maximum length of the femur in the archaeological material to calculate the stature of the individuals. We collected the same vertebral measurements from the MRI material, using clinical workstations (neaView radiology, Neagen, Oulu, Finland) and a linear measurement tool (accuracy 0.1 mm).

As our archaeological vertebral material was collected by two researchers, NK and TV, we decided to test for interobserver error with specimens that had been measured by both. We ran Intraclass correlation coefficient (ICC) and obtained coefficients over 0.9 for all measurements, indicating high reliability. The fact that the modern human data were collected using MRI could also have affected the accuracy of the measurements. However, our previous study^18^ demonstrated that the MRI-based measurements are equivalent to those taken using osteometric callipers.

From the collected measurements, we calculated the mean craniocaudal (CC) vertebral height as the mean of the anterior and posterior height. We chose mean height as we wanted to study the changes in vertebral size and dimensions rather than how vertebral wedging had changed. We also calculated the vertebral cross-section area (CSA) using the formula: *π • a • b*, where ‘*a*’ is the maximum vertebral ML width/2 and ‘*b*’ is vertebral AP depth/2, and we calculated the volume of the vertebral body as a cylinder, using the formula: CSA • mean CC height. The ratio of ML width to AP depth was calculated as ML width/AP depth. Stature estimations for the archaeological individuals were calculated from the maximum length of the femur, using the regression formula of Ruff *et al*.^47^. For three individuals, we took stature from the osteological analysis^20,21^, because their femur lengths were not available. Sex data were partly available to us from the osteological analysis^20,21,48,49^ and partly assessed by one of the authors using standard osteological techniques.

IBM SPSS Statistics version 25 (IBM Corporation, Armonk, NY, USA) was used to perform the statistical analysis. P values of <0.05 were considered statistically significant. First, after ensuring the data followed a fairly normal distribution, we calculated the means and standard deviations (SDs) of stature and each vertebral dimension for each sample. Then, we compared the modern samples to the archaeological sample using general linear modelling (GLM). In the GLM models, we used stature and vertebral dimensions as continuous outcome variables, each separately; the ‘sample’ variable (i.e. archaeological/modern 20y/modern 30y/modern 46y) acted as the explanatory variable. The archaeological sample was chosen as the reference category to which the modern samples were compared. Where appropriate, we also re-ran the analyses by including the ‘stature’ variable in the models as a continuous covariate. As stature is strongly associated vertebral dimensions^33^, and it has clearly increased over time^36^, we considered the adjustment necessary in order to rule out its confounding effect on the results. All the models were stratified by sex due to the strong sex discrepancy in the vertebral dimensions^11^. The results of these analyses are presented in Table 1. As the results include the actual dimensions (in mm) in addition to the height-adjusted dimensions, for easier separation, we will refer to the actual dimensions as unadjusted.

## Data Availability

The archaeological data are not publicly available, neither is the modern human data due to local privacy regulations. However, both are available from the corresponding author on reasonable request.
